# The signature of pyroptosis-related gene prognostic and immune microenvironment in adrenocortical carcinoma

**DOI:** 10.3389/fmolb.2023.1131402

**Published:** 2023-02-24

**Authors:** Jun Gao, Dai Wang, Qingping Yang, Mengjie Tang, Jiayi Du, Leye He, Wei Liu

**Affiliations:** ^1^ Department of Urology, The Third Xiangya Hospital, Central South University, Changsha, China; ^2^ Xiangya School of Pharmacy, Central South University, Changsha, China; ^3^ Department of Pharmacy, Zunyi Medical University, Zunyi, China; ^4^ Department of Pathology, Hunan Cancer Hospital, The Affiliated Cancer Hospital of Xiangya School of Medicine, Central South University, Changsha, China; ^5^ Department of Pharmacy, Fuqing City Hospital of Fujian, Fuqing, China; ^6^ Department of Pharmacy, The Third Xiangya Hospital, Central South University, Changsha, China

**Keywords:** pyroptosis, ACC, prognostic gene signature, immune infiltrates, immune checkpoints

## Abstract

Adrenocortical carcinoma (ACC) has a low incidence but a poor prognosis. And ACC has complex clinical manifestations and limited treatment. Pyroptosis has a dual character and has both positive and negative effects on cancer. However, the role of pyroptosis-related genes (PRGs) in ACC and the impact on ACC progression remains unelucidated. This study performed systematic bioinformatics analysis and basic experimental validation to enable the establishment of prognostic models and demonstrate levels of immune infiltration. Pearson’s correlation analysis was used to assess the association of PRGs with tumor immune infiltration, tumor mutation burden (TMB), microsatellite instability (MSI), and immune checkpoints. There 4 PRGs were upregulated, and 25 PRGs were downregulated in ACC. At the same time, we analyzed and reviewed the genetic mutation variation landscape of PRGs. Functional enrichment analysis was also performed to clarify the function of PRGs. Pyroptosis, the inflammatory response, the Toll-like receptor signaling pathway, and the NOD-like receptor signaling pathway are the functions and pathways mainly involved and exerted effects by these 33 PRGs. The results of the prognosis analysis revealed high expression of CASP3, CASP9, GSDMB, GSDMD, NLRC4, PRKACA, and SCAF11 caused a poor survival rate for ACC patients. The above seven PRGs were screened by the optimal λ value of LASSO Cox analysis, and the five selected genes (CASP3, CASP9, GSDMB, GSDMD, NLRC4) were involved in constructing a prognostic PRGs model which enables the overall survival in ACC patients can be predicted with moderate to high accuracy. Prognostic PRGs, especially CASP9, which is the independent factor of ACC prognosis, may be closely correlated with immune-cell infiltration, tumor mutation burden, microsatellite instability, and immune checkpoints. Quantitative Real-Time PCR (qRT-PCR), Western blot and immunohistochemical were performed to validate the mRNA expression levels of CASP9 in adjacent normal tissues and ACC tissues. According to the result of immune checkpoints analysis, NLRC4 and GSDMB may be identified as potential therapeutic targets. In conclusion, we established a prognostic model of PRG characteristics in ACC and analyzed the relationship between PRGs and immune infiltration. Through our study, it may be helpful to find the mechanism of pyroptosis in ACC.

## 1 Introduction

Adrenocortical carcinoma (ACC), also known as adrenocortical adenocarcinoma or cortical carcinoma, is a malignant epithelial tumor originating from adrenal cortical cells. Although the incidence of ACC is low, about 0.7-2 cases per 100,000 people per year, the clinical manifestations of ACC are complex and diverse. ACC is diagnosed late with limited treatment and poor prognosis, and in advanced disease, 5-year survival is less than 15%, with a high recurrence rate even after radical surgery (Pittaway and Guasti, 2019). Although many papers have suggested some potential therapeutic targets for ACC and biomarkers that can predict the prognosis of ACC, clinical practice has not been carried out. Therefore, it is necessary to analyze the prognostic gene signature of ACC.

Pyroptosis is closely related to tumor progression and has both positive and negative effects on tumors. On the one hand, pyroptosis can inhibit tumor development as an innate immune mechanism. On the other hand, as a pro-inflammatory cell death mode, pyroptosis provides a suitable tumor microenvironment (Yu et al., 2021). As one of the research hotspots in recent years, many researchers have analyzed the relationship between pyroptosis and tumors. Researchers have found that pyroptosis is closely related to the occurrence and development of many cancers. For example, a recent study suggested PAGsPI and IAGsPI as potential biomarkers for predicting prognosis in patients with renal clear cell carcinoma (Lin et al., 2022). The characteristics of the pyroptosis-related prognostic gene and the correlation regulation axis in lung adenocarcinoma were also identified (Lin et al., 2021). A model based on PRGs can be used to predict the prognosis of colon adenocarcinoma and can be well validated by the external cohort (Luo et al., 2021). In the study of generalized cancer, researchers found that most of the pyroptosis genes were abnormally expressed in different cancers through differences in CNV (copy-number variant) frequency and DNA methylation levels (Liu et al., 2022). Moreover, GSDMD, a member of the gasdermin family, was differentially expressed in the vast majority of cancer and could be used as a prognostic marker in ACC (Qiu et al., 2021).

However, it remains unelucidated what role pyroptosis plays in ACC, and these issues will be explored and discussed in this study. Moreover, we hope to search for potential biomarkers and prognostic factors by exploring public databases to prompt expression, mutation, prognosis, and immune infiltration.

## 2 Methods and materials

### 2.1 Datasets and preprocessing

We downloaded the RNA-sequencing (RNA-seq) data and the corresponding clinical information from The Cancer Genome Atlas (TCGA) database and the University of California, Santa Cruz (UCSC) Xena website (https://xenabrowser.net/datapages/). Supplementary Table S1 presents the clinical information of ACC patients. CNV data and somatic datasets for ACC were also obtained using the TCGA database. Data analysis was performed by R (version 4.0.3) and R Bioconductor packages. The expression data were normalized to transcripts per kilobase million (TPM) values before further analysis.

### 2.2 The different expressions of PRGs

33 PRGs were obtained from previous studies (Latz et al., 2013; Ye et al., 2021), as shown in Supplementary Table S2. The expression differences of PRGs in ACC and normal tissues were determined using the R package “ggplot2.” Subsequently, the search tool for the retrieval of interacting genes (STRING) was used to construct protein-protein interaction (PPI) networks for 33 PRGs to retrieve interacting genes.

### 2.3 Mutational analysis of PRGs

The frequency of gene mutation and oncoplot waterfall plots for 33 PRGs in ACC patients were exported by the “maftools” package. The R package “gaia” was used for data processing to draw chromosome segment maps and mark CNV changes of 33 PRGS on 23 chromosomes. The CNV of the target gene was visualized using the “ggplot2” package in R.

### 2.4 Functional enrichment analysis of PRGs

The R package “clusterprofiler” was utilized to perform Gene Ontology (GO), including the biological process (BP), cellular component (CC), and molecular function (MF) categories. And the Kyoto Encyclopedia of Genes and Genomes (KEGG) analysis was also conducted by this package.

### 2.5 Screening PRGs with prognostic value

To evaluate the prognostic significance of the PRGs, we used the Cox regression analysis to perform. The *p*-values and hazard ratios (HRs) with 95% confidence intervals (CIs) of the Kaplan-Meier curves were obtained by log-rank tests and univariate Cox proportional hazard regression. PRGs will be screened by prognostic value and those with significant prognostic value will be selected for further analysis.

### 2.6 Construction of the pyroptosis-related gene prognostic model

Based on these prognostic PRGs, a prognostic model was constructed using LASSO Cox regression analysis. TCGA ACC patients were divided into low-risk and high-risk subgroups based on median risk score, and the overall survival (OS) time of the two subgroups was compared by Kaplan-Meier analysis. Time receiver-operating characteristic (ROC) analysis was used to determine the accuracy of the prediction model.

### 2.7 Construction of a predictive nomogram

Taking clinical features into account, a predictive nomogram was developed for prediction. Univariate and multivariate Cox regressions were first performed, and forest plots were used to display each variable (*p-*value, HR, and 95% CI) through the “forestplot” package. Based on the results of multivariate Cox proportional risk analysis, the “rms” package was used to construct a rosette to predict the total recurrence rate at 1-year, 3-year, and 5-year.

### 2.8 Immune infiltration analysis

Subsequently, we used the Tumor Immune Estimation Resource (TIMER, https://cistrome.shinyapps.io/timer/), a web portal for comprehensive analysis of tumor-infiltrating immune cells, to analyze the association between prognostic PRGs and immune infiltration. The correlation between pyroptosis-related prognostic genes and the level of immune infiltration in ACC can be visually shown by the “Gene” module of TIMER. Spearman’s correlation analysis was performed for TMB and MSI analysis to calculate the correlation between gene expression and TMB and MSI scores. Results with a *p*-value less than 0.05 was considered statistically significant.

### 2.9 Immune-checkpoints analysis

We selected SIGLEC15, TIGIT, CTLA4, CD274, HAVCR2, LAG3, PDCD1, and PDCD1LG2 as immune-checkpoint-relevant transcripts and extracted the expression data of these eight genes. To assess the expression of the immune checkpoints and co-expression of prognostic PRGs with these immune checkpoints.

### 2.10 Tissue samples

During March and July 2022, we collected 12 pairs of ACC tissues and adjacent normal tissues from the Third Xiangya Hospital of Central South University. These tissues were used to detect CASP9 expression levels by qRT-PCR, Western blot and immunohistochemistry. The Ethics Committee of the Third Xiangya Hospital of Central South University has approved the study. The approval number is I-22188.

### 2.11 qRT-PCR

To lyse cells, 1 mL Trizol reagent (TaKaRa, Japan) was added to the sample tissue according to the actual manufacturer’s instructions and incubated on a shaker for 15 min at room temperature. Total RNA was subsequently extracted from target samples. One microgram of RNA was reverse transcribed into cDNA using the Revert Aid First Strand cDNA Synthesis Kit (Thermo, United States). Quantitative RT-PCR was then performed with Pro Taq HS Premix Probe qPCR Kit (Accurate, Hunan, China). The GAPDH gene was used as an endogenous control gene for normalizing the expression of target genes. Primers used in this study included CASP9 (forward 5′-CTG​TCT​ACG​GCA​CAC​AGA​TGG​AT-3′, reverse 5′- GGG​ACT​CGT​CTT​CAG​GGG​AA-3′), GAPDH (5′-CAG​GAG​GCA​TTG​CTG​AT-3′, 5′-GAA​GGC​TGG​GGC​TCA​TTT-3′).

### 2.12 Western blot

Tissues were lysed with RIPA lysis buffer containing protease and phosphatase inhibitors (Thermo Fisher Scientific, United States), and protein lysates were resolved by SDS-PAGE gels (Thermo Fisher Scientific, United States), blotted onto PVDF membranes (Roche, Switzerland) for analysis, and incubated overnight at 4°C with anti-CASP9 antibody (1:1,000 dilution; proteintech, China) and anti-ACTB antibody (1:2000 dilution; Cell Signaling Technology, United States). Data analysis was performed using Image J software.

### 2.13 Immunohistochemical staining

The tissues were sectioned and embedded in paraffin. Sections were incubated overnight at 4°C with anti-CASP9 antibody (1:100 dilution; proteintech, China). Slides were washed with phosphate-buffered saline (PBS) and incubated with a goat anti-rabbit IgG secondary antibody conjugated with fluorescein isothiocyanate (ZSDB-BIO, China) for 30 min with washed slides. After washing with PBS, they were incubated with an antifade reagent (Invitrogen, United States). Staining was visualized to determine protein expression levels using an Olympus CX41 fluorescence microscope (Olympus, Japan). The analysis results were performed using Image J software.

## 3 Results

### 3.1 The different expression of PRGs of ACC

**FIGURE 1:**
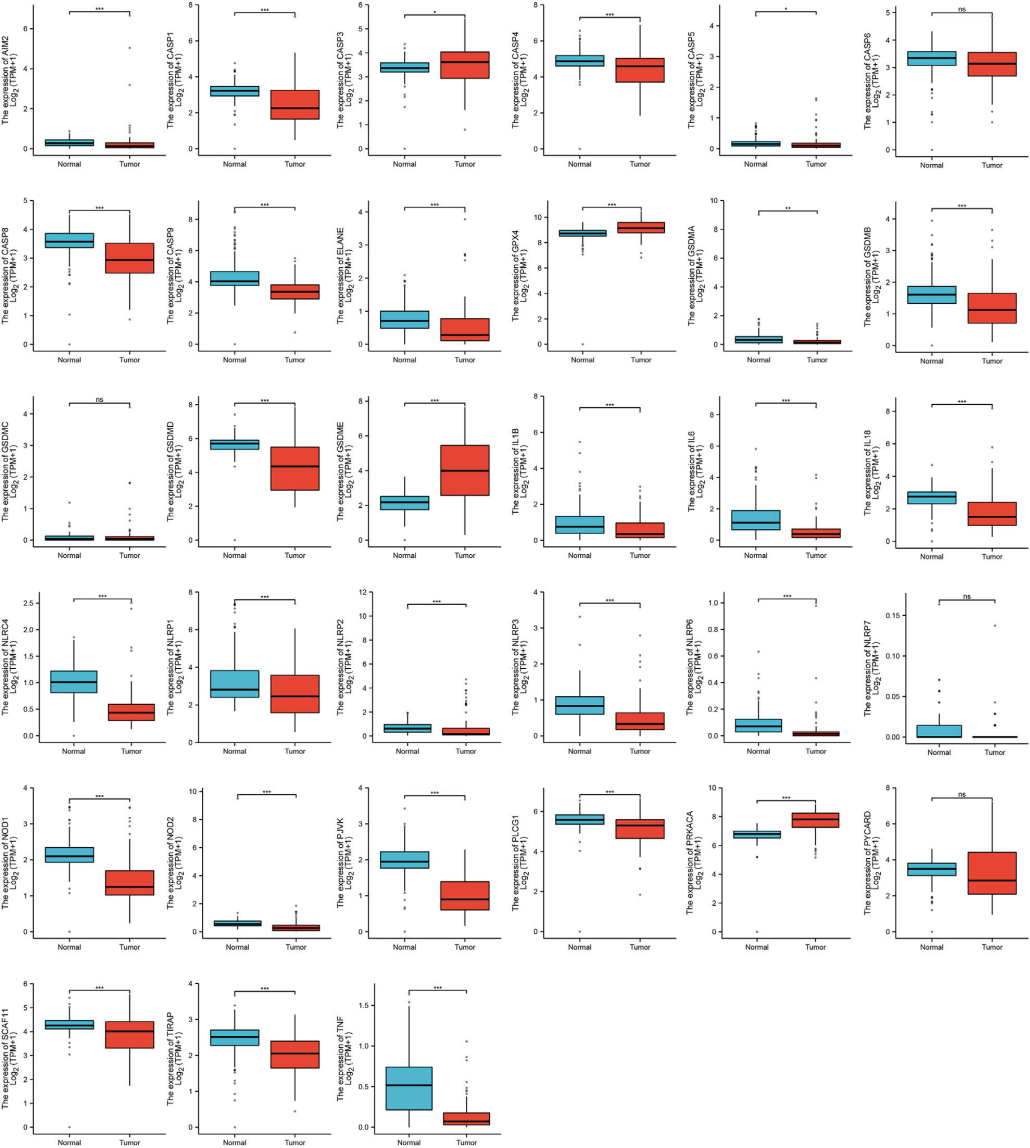
The expression of PRGs in ACC. [*p < 0.05, **p < 0.01, ***p < 0.001, asterisks (*) stand for significance levels].

Firstly, we analyze the different expressions of the 33 PRGs in ACC and the normal adrenal cortex by the UCSC Xena. The results of the analysis of four genes were not meaningful (CASP6, GSDMC, NLRP7, and PYCARD), except for 29 PRGs were up–regulated or down–regulated in ACC (Figure 1). Compared with normal tissues, the expression of CASP3, GPX4, GSDME, and PRKACA was increased, while the expression of AIM2, CASP1, CASP4, CASP5, CASP8, CASP9, ELANE, GSDMA, GSDMB, GSDMD, IL1B, IL6, IL18, NLRC4, NLRP1, NLRP2, NLRP3, NLRP6, NOD1, NOD2, PJVK, PLCG1, SCAF11, TIRAP, and TNF was decreased in ACC. We also constructed a PPI analysis to find the interaction of these PRGs (the minimum required interaction score of 0.9). The result of the PPI indicates that CASP1, CASP5, CASP8, NLRP1, NLRP3, and PYCARD were hub genes (Supplementary Figure S1A). Supplementary Figure S1B shows the correlation network containing all PRGs.

### 3.2 The gene mutation of PRGs in ACC

**FIGURE 2:**
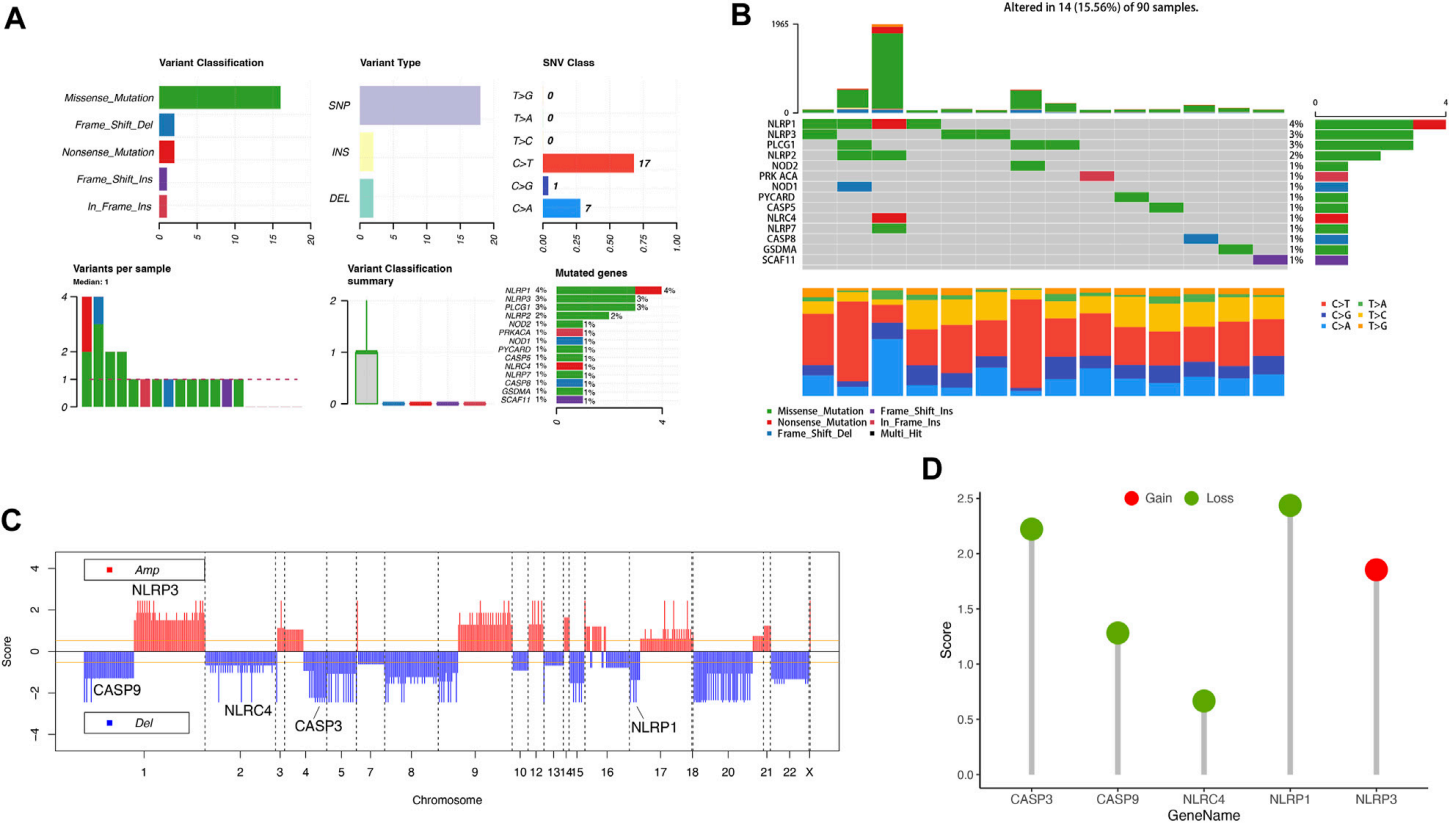
Landscape of genetic variation of PRGs in ACC. **(A, B)** The mutation of frequency and classification of 33 PRGs in ACC. **(C)** The location of CNV alteration of 33 PRGs on 23 chromosomes in the ACC cohort. **(D)** The CNV variation frequency of 33 PRGs in the ACC cohort. The height of the column represented the alteration frequency.

We performed a summary of CNV and somatic mutations of 33 PRGs in ACC. From Figures 2A, B, we confirmed that gene mutation occurred in 14 of 90 (15.56%) ACC samples. The most common variant classification is Missense mutation, and the most common variant type is SNPs (single nucleotide polymorphisms), and C>T ranked as the top SNV (single nucleotide variants) class (Figure 2A). We also found the first three mutation frequency genes in Figure 2B: NLRP1, NLRP3, and PLCG1. The location of CNV alterations of these 33 PRGs on chromosomes was presented in Figure 2C. We also investigated CNV alteration frequency, revealing that the CNV deletion frequencies of CASP3, CASP9, NLRC4, and NLRP1 were widespread (Figure 2D).

### 3.3 Functional enrichment analysis of PRGs

**FIGURE 3:**
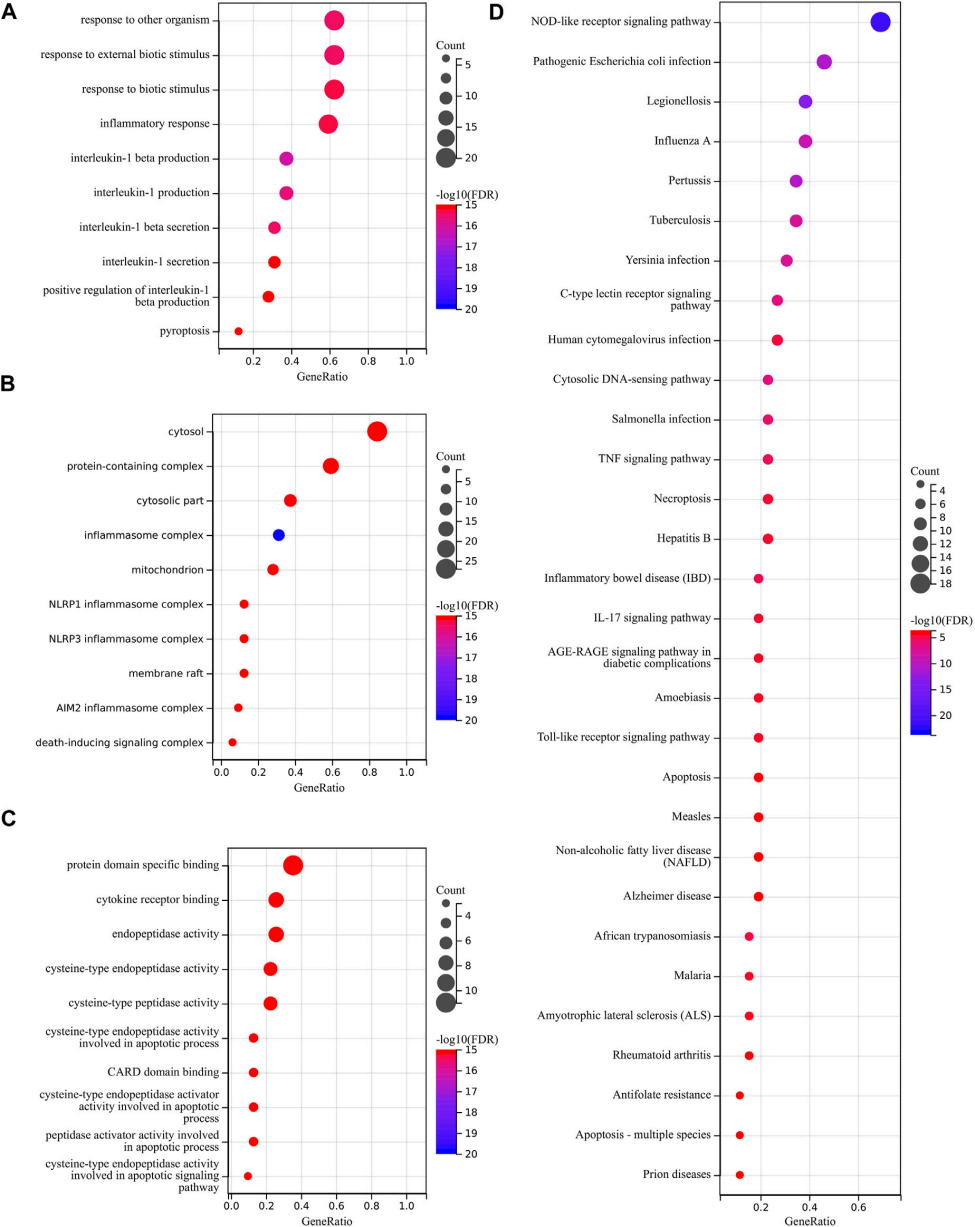
Functional enrichment analysis of PRGs. **(A–C)** The enriched item in gene ontology analysis. **(D)** The enriched item in Kyoto Encyclopedia of Genes and Genomes analysis.

We used GO and KEGG databases for functional pathway enrichment analysis to reveal the function of PRGs. These 33 PRGs were mainly involved in response to other organisms, inflammatory response, interleukin-1 beta production, pyroptosis, cytosol, inflammasome complex, cytokine receptor binding, and cysteine-type peptidase activity in GO analysis (Figures 3A–C). Otherwise, the KEGG analysis has shown that 33 PRGs were mainly involved in the NOD-like receptor signaling pathway, human cytomegalovirus infection, cytosolic DNA-sensing pathway, TNF signaling pathway, necroptosis, and Toll-like receptor signaling pathway (Figure 3D).

### 3.4 Construct the pyroptosis-related prognostic gene model

**FIGURE 4:**
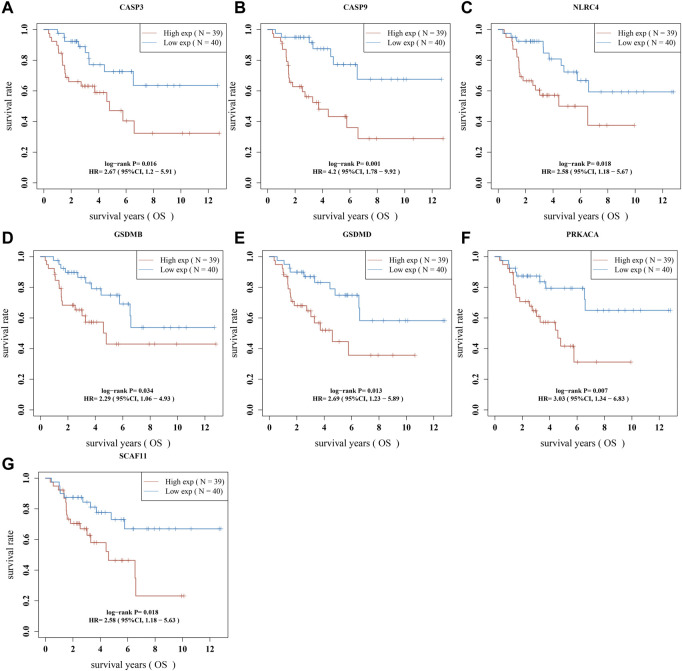
The prognostic value of PRGs in ACC. The Kaplan–Meier survival curves of **(A)** CASP3, **(B)** CASP9, **(C)** NLRC4, **(D)** GSDMB, **(E)** GSDMD, **(F)** PRKACA, and **(G)** SCAF11 in ACC patients in the high-/low-expression group.

**FIGURE 5:**
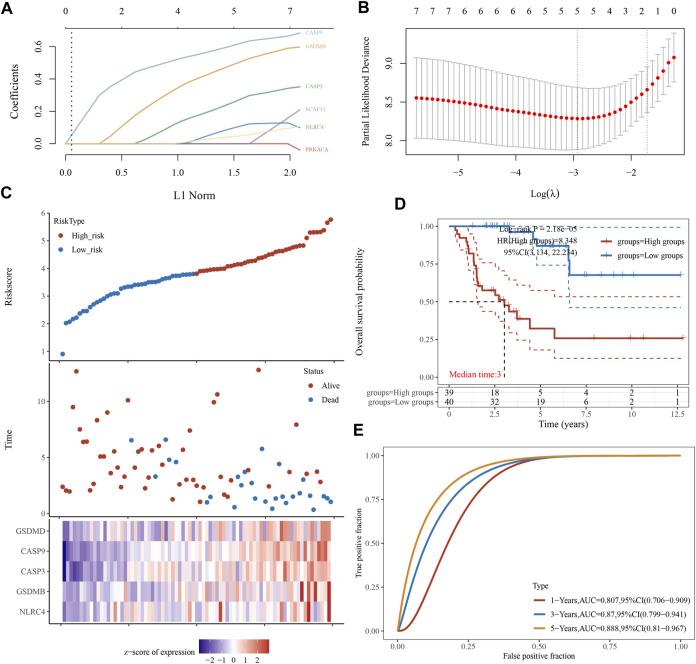
Construction of a prognostic PRGs model. **(A)** LASSO coefficient profiles of the seven PRGs. **(B)** Plots of the ten-fold cross-validation error rates. **(C)** Distribution of risk score, survival status, and the expression of five prognostic PRGs in ACC. **(D, E)** Overall survival curves for ACC patients in the high-/low-risk group and the ROC curve for measuring the predictive value.

We performed a univariate Cox regression analysis to find PRGs with a prognostic value, and seven PRGs were screened. The Kaplan-Meier survival curves were shown in Figure 4. The results indicated that ACC patients with high expression of CASP3 (Figure 4A, *p* = 0.016), CASP9 (Figure 4B, *p* = 0.001), NLRC4 (Figure 4C, *p* = 0.018), GSDMB (Figure 4D, *p* = 0.034), GSDMD (Figure 4E, *p* = 0.013), PRKACA (Figure 4F, *p* = 0.007), and SCAF11 (Figure 4G, *p* = 0.018) have a poor survival rate. A five-gene model was constructed according to the optimum λ value obtained by LASSO Cox analysis (Figures 5A, B). The risk score was calculated as follows: (0.0812) * NLRC4 + (0.4678) * GSDMB + (0.2337) * CASP3 + (0.5923) * CASP9 + (0.0383) * GSDMD. The ACC patients were divided into two groups based on the risk score. Figure 5C indicated that patients are at increased risk of death and survival time decreased as the risk score increases. Kaplan-Meier curves showed the ACC patients in the high groups had a lower probability of overall survival than patients in the low groups (median time = 3 years, *p* = 2.18e-05; Figure 5D), with AUCs of 0.807, 0.87, and 0.888 in the 1-year, 3-year, and 5-year ROC curves, respectively (Figure 5E).

### 3.5 Constructing the predictive nomogram

**FIGURE 6:**
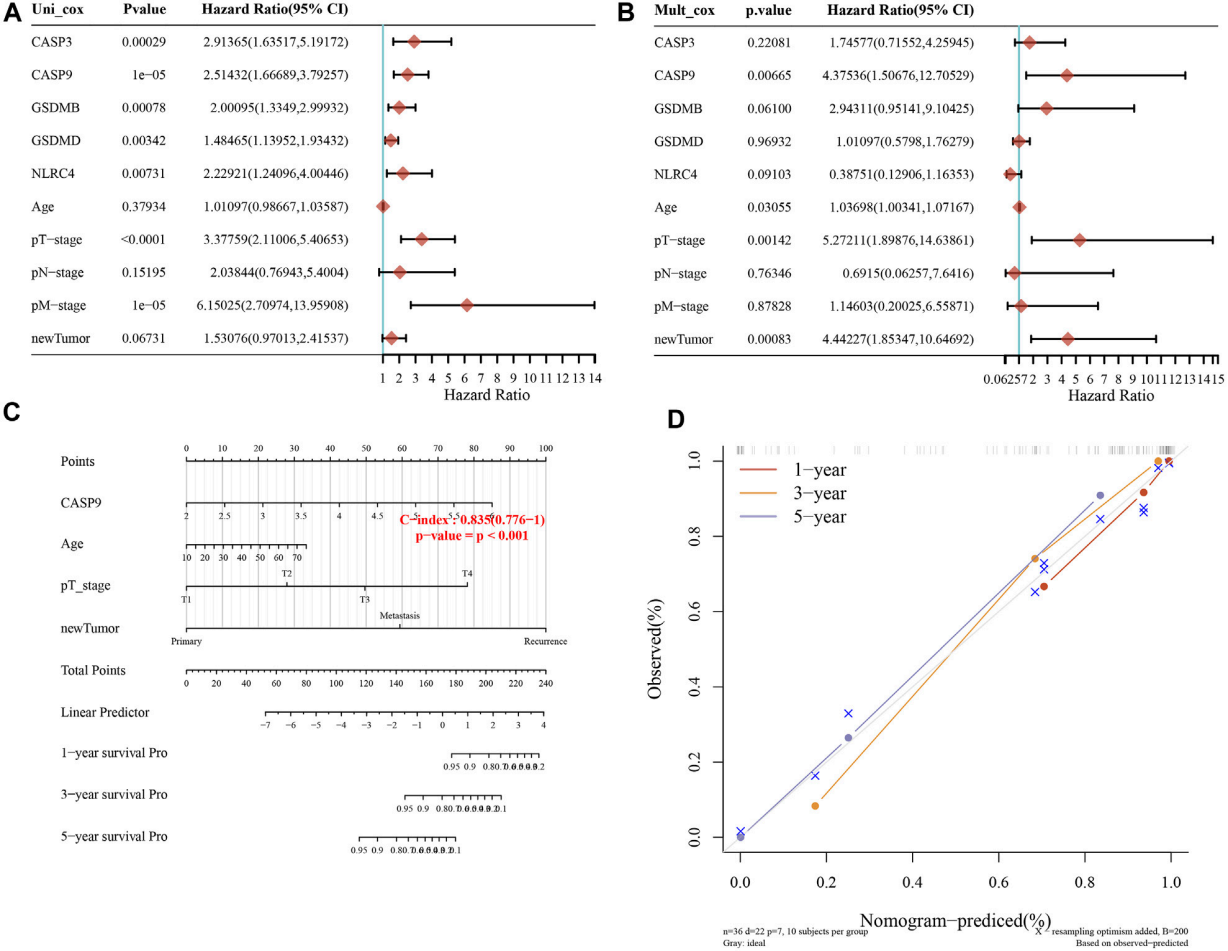
Constructing the predictive nomogram. **(A, B)** Hazard ratio and *p*-value of the constituents involved in univariate and multivariate Cox regression considering clinical parameters and five prognostic PRGs in ACC. **(C)** The 1-year, 3-year, and 5-year overall survival rates of ACC patients were predicted by the nomogram. **(D)** Calibration curve for the overall survival nomogram model in the discovery group.

A predictive nomogram was constructed to predict the survival probability. These five PRGs and the clinicopathologic features were considered in the nomogram. According to univariate and multivariate analyses, the expression of CASP9, newTumor, and the pT stage were identified as independent factors affecting the prognosis of ACC patients (Figures 6A, B). The significant variables were screened based on the univariate and multivariable regression analysis results to construct the nomogram. Apparently, the staging of CASP9, newTumor, and pT stage can be significant and accurately predict the survival time of patients (C-index represents the accuracy of prediction, and the *p-*value of Figure 6C represents the significance of the nomogram prediction model). Predicted nomograms suggest relatively good prediction of 1-year, 3-year, and 5-year overall survival rates compared to ideal models in the entire cohort (Figure 6D).

### 3.6 The correlation between tumor immune infiltration and PRGs in ACC

**FIGURE 7:**
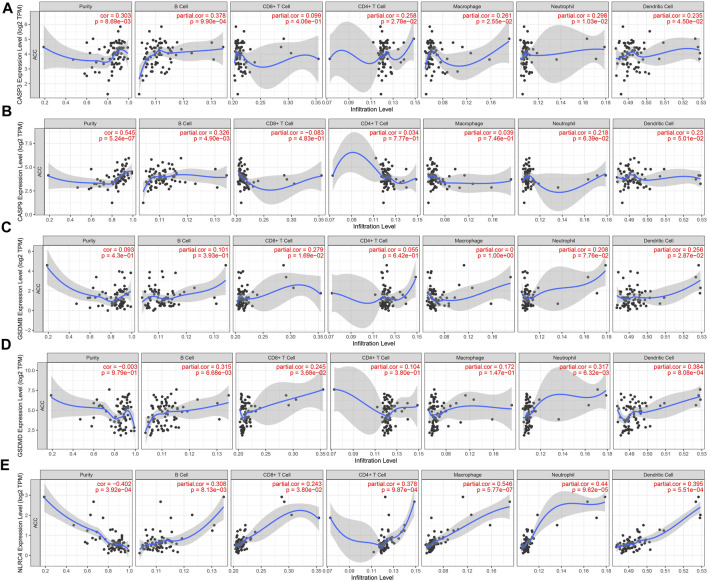
The correlation between five prognostic PRGs and immune infiltration. The association between the abundance of immune cells and the expression of CASP3 **(A)**, CASP9 **(B)**, GSDMB **(C)**, GSDMD **(D)**, and NLRC4 **(E)** in ACC.

In the development of the tumor-immune microenvironment, pyroptosis plays a very important role. The correlation between tumor immune infiltration and PRGs in ACC was identified by the TIMER database. The expression of CASP3 was positively associated with the abundance of B cells (*p* = 9.90e-04), CD4 + T cells (*p* = 2.78e-02), macrophages (*p* = 2.55e-02), neutrophils (*p* = 1.03e-02), and dendritic cells (*p* = 4.50e-02) (Figure 7A). And the correlation between CASP9 expression and the immune infiltration level of B cells (*p* = 4.90e-03, Figure 7B) was also positive. GSDMB expression showed a positive relation with CD8 + T cells (Figure 7C, *p* = 1.69e-02), and dendritic cells (*p* = 2.87e-02). Moreover, GSDMD expression was positively related to B cells (*p* = 6.68e-03), CD8 + T cells (*p* = 3.68e-02), neutrophils (*p* = 6.32e-03), and dendritic cells (*p* = 8.08e-04) (Figure 7D). A positive correlation between NLRC4 and B cells (*p* = 8.13e-03), CD8 + T cells (*p* = 3.80e-02), CD4 + T cells (*p* = 9.87e-04), macrophages (*p* = 5.77e-07), neutrophils (*p* = 9.62e-05), dendritic cells (*p* = 5.51e-04) (Figure 7E) was demonstrated. These results proved the correlation between PRGs and tumor immune infiltration.

### 3.7 Analyze the association between PRGs and TMB, MSI, and drug-sensitivity

**FIGURE 8:**
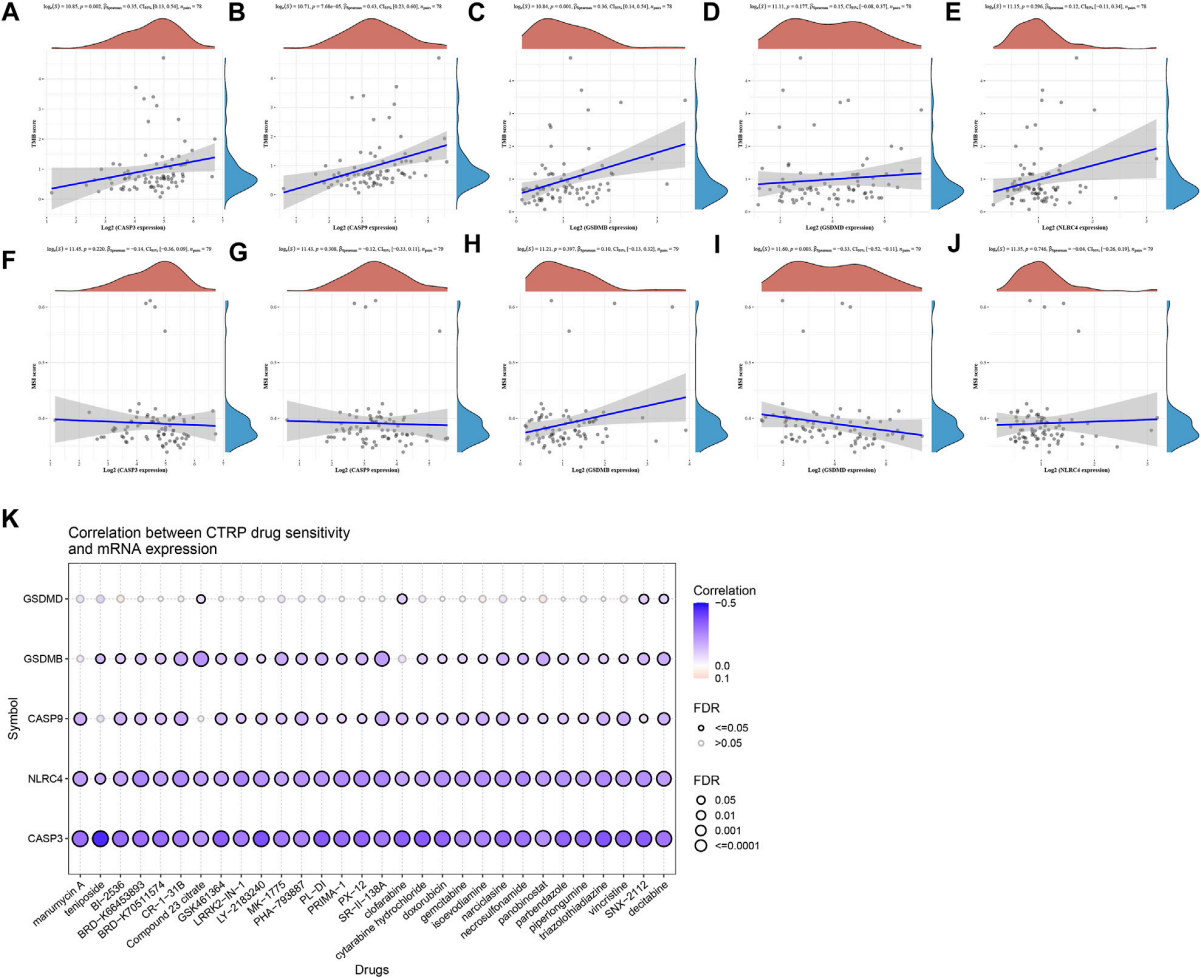
TMB, MSI, drug-sensitivity analysis of PRGs in ACC. **(A–E)** Correlation between five prognostic PRGs and TMB. **(F–J)** Correlation between five prognostic PRGs and MSI. **(K)** Drug-sensitivity analysis of five prognostic PRGs in ACC.

TMB and MSI were closely related to immunotherapy for cancer (Chang et al., 2018; Liu et al., 2019; Samstein et al., 2019). TMB was recognized as a biomarker that can predict immunotherapy effects. And MSI can also be suggested as a predictive biomarker for cancer immunotherapy. We have clarified that these prognostic PRGs were closely related to tumor immune infiltration. The analysis of the correlation between PRGs and TMB and MSI in ACC aimed to reveal whether the PRGs play as a biomarker for drug screening. TMB was positively correlated to CASP3 (Figure 8A, *p* = 0.002), CASP9 (Figure 8B, *p* = 7.68e-05), and GSDMB (Figure 8C, *p* = 0.001); There is no significant correlation between TMB and GSDMD (Figure 8D), and NLRC4 (Figure 8E). The results of MSI analysis suggested that MSI was negatively correlated to GSDMD (Figure 8I, *p* = 0.003); Other PRGs did not show a significant correlation with MSI (Figures 8F–H, J). The drug sensitivity analysis revealed that CASP3, CASP9, GSDMB, GSDMD, and NLRC4 were associated with multiple anticancer drug efficacy, which suggested that pyroptosis genes may be involved in pan-cancer signaling pathways and influence anticancer drug efficacy in the cancer therapeutic response portal database (Figure 8K).

### 3.8 Immune checkpoints analysis of ACC

**FIGURE 9:**
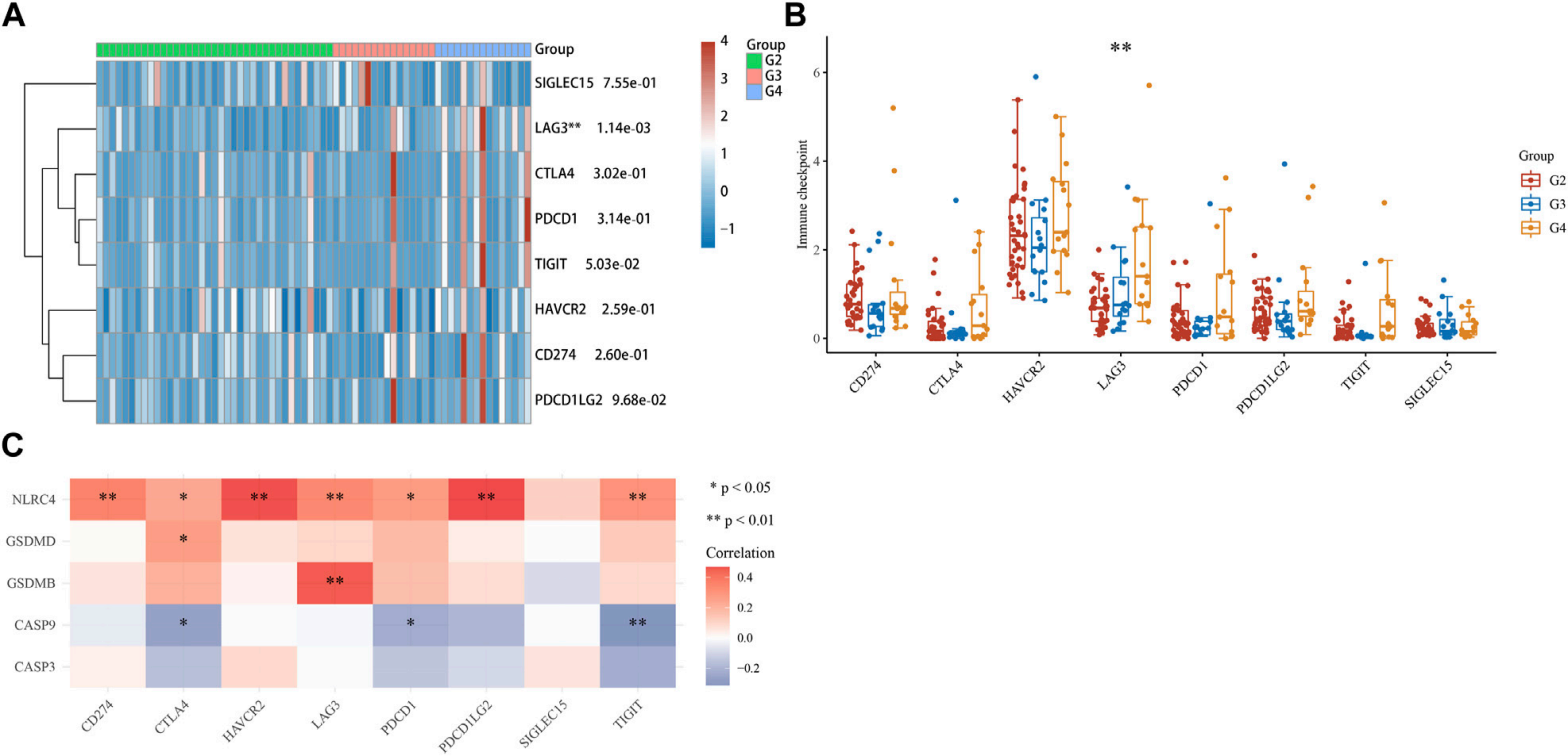
Immune checkpoints analysis. **(A, B)** Different expressions of immune checkpoints in G2, G3, and G4 of ACC. (*p < 0.05, **p < 0.01, ***p < 0.001, asterisks (*) stand for significance levels.). **(C)** The correlation analysis between five prognostic PRGs and immune checkpoints.

As the samples of G1 patients were insufficient to participate in the analysis, the expression of immune checkpoints in the G2, G3, and G4 stages of ACC was analyzed, including SIGLEC15, TIGIT, CTLA4, CD274, HAVCR2, LAG3, PDCD1, and PDCD1LG2. Results revealed the expression of LAG3 (*p* = 1.14e-03) increased sequentially in G2, G3, and G4 stages (Figures 9A, B). Moreover, we analyzed the correlation between five prognostic genes and immune checkpoints and found that NLRC4 and GSDMB were positively correlated with LAG3, which means NLRC4 and GSDMB could be a potential immunotherapeutic target of ACC (Figure 9C).

### 3.9 Validation of mRNA and protein expression levels of CASP9 in ACC

**FIGURE 10:**
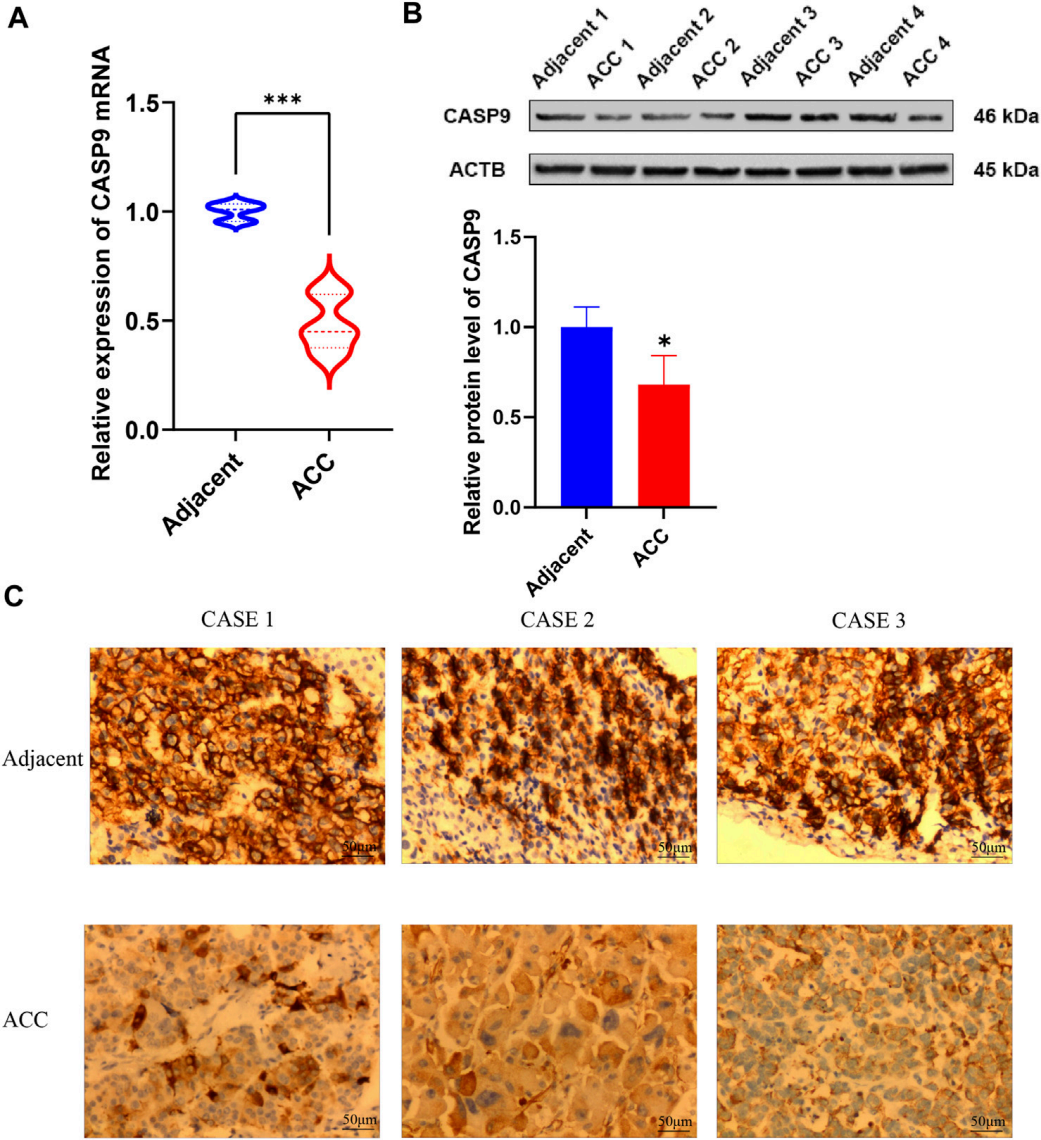
Validation of mRNA and protein expression levels of CASP9 in ACC. **(A)** Comparison of mRNA expression levels of CASP9 by qRT-PCR in 12 paired ACC tissues and adjacent normal tissue samples. **(B)** The Western blot analyses. **(C)** The immunohistochemical staining in 12 ACC tissues and adjacent normal tissues.

Quantitative real-time PCR (qRT-PCR) was performed to validate CASP9 expression levels in ACC and adjacent normal tissues. The results showed the expression level of CASP9 in ACC was significantly lower than that in adjacent non-cancerous tissues (Figure 10A), which was consistent with the results of gene difference analysis. The protein expression level of CASP9 was subsequently determined by Western blotting and immunohistochemical staining, and the results were consistent with the expression level of mRNA (Figures 10B, C).

## 4 Discussion

Firstly, we revealed the expression and gene mutation of PRGs in ACC. The results presented the expression of CASP3, GPX4, GSDME, and PRKACA was increased, while the expression of AIM2, CASP1, CASP4, CASP5, CASP8, CASP9, ELANE, GSDMA, GSDMB, GSDMD, IL1B, IL6, IL18, NLRC4, NLRP1, NLRP2, NLRP3, NLRP6, NOD1, NOD2, PJVK, PLCG1, SCAF11, TIRAP, and TNF was decreased in ACC. Prior results have revealed that GSDMD expression was closely related to ACC and could be used as a prognostic marker of cancer (Qiu et al., 2021). Moreover, the top three genes in mutation frequency were NLRP1 (4%), NLRP3 (3%), and PLCG (3%), and this result may serve as a potential risk screening basis. Alternatively, NLRP1 and NLRP3 have both somatic mutations and copy number variations that may play a role in exploring potential therapeutic targets in ACC.

In functional enrichment analysis, the NOD-like receptor signaling pathway, inflammatory response, TNF signaling pathway, Toll-like receptor signaling pathway, the regulation of the inflammatory response, and pyroptosis are the functions and pathways mainly involved and exerted effects by these 33 PRGs. Most of these functions or pathways participated in ACC’s occurrence and development process. Zhang (Zhang et al., 2022) has discovered that PIRK2 could cause the occurrence and development of ACC. And the research of Waldemar Kanczkowski (Kanczkowski et al., 2010) reveals that the expression of TLR4 in ACC was significantly reduced, and the TLR4 signaling pathway was inactivated. By studying these functions or pathways, we may be able to find more effective treatments for ACC.

We performed a univariate Cox regression analysis, and seven PRGs (CASP3, CASP9, GSDMB, GSDMD, NLRC4, SCAF11, and PRKACA) with their Kaplan-Meier survival curves were obtained. The prognostic analysis suggested that ACC patients with high expression of CASP3, CASP9, GSDMB, GSDMD, NLRC4, PRKACA, and SCAF11 would have a low survival rate. The prognostic model based on five PRGs (CASP3, CASP9, GSDMB, GSDMD, and NLRC4) was constructed by LASSO Cox regression analysis, and this model enables overall survival to be predicted with moderate to high accuracy. We also find three independent factors affecting the prognosis of ACC patients: CASP9, newTumor, and the pT stage. Interestingly, CASP9 has already been known as a multimodal therapeutic target with diverse cellular expression in human disease, and CASP9 polymorphisms have been linked with various cancers, neurological disorders, autoimmune pathologies, and lumbar disc disease (Avrutsky and Troy, 2021). Predicted nomograms suggest relatively good prediction of 1-year, 3-year, and 5-year overall survival rates compared to ideal models in the entire cohort. Several studies have identified some prognostic signatures for ACC patients. Yuan (Yuan et al., 2022) verified 13 immune-related genes as prognostic characteristics of ACC. Chen (Chen et al., 2021) established prognostic features of ferroptosis in ACC. In addition, m6A-related risk characteristics are conducive to the prognostic analysis and can affect the immune microenvironment in ACC (Xu et al., 2021). Through our study, we identified the features of pyroptosis-related prognostic genes in ACC, which provides more choices for prognostic prediction of ACC.

Are there other genes that are also of research value? We found that PRKACA is one of the genetic signatures in our study. Previous studies have suggested that PRKACA plays an important role in the development of some tumors. PRKACA gene defects can lead to adrenal cortical carcinoma (Berthon et al., 2015), but PRKACA is not directly related to pyrosis. PRKACA encodes one of the catalytic subunits of protein kinase A (PKA), whose camp-dependent phosphorylation of proteins is important for many cellular processes, including differentiation, proliferation, and apoptosis. PRKACA is also involved in gene fusion, and fusion genes containing PRKACA have been detected in fibrolamellar carcinoma and intraductal eosinophilic papillary tumors of the pancreas (Rahi et al., 2022). In addition, overexpressed PRKACA inhibits RNA virus-induced IFN-β promoter activation and IFNB1 gene transcription (Yan, 2017). These studies demonstrate that PRKACA has a broad effect. However, relevant studies on the role PRKACA plays in pyroptosis are limited. Whether PRKACA plays a role during pyroptosis in ACC remains to be confirmed *in vitro* and *in vivo* studies.

The results of immune infiltration analysis suggested these five PRGs are significantly associated with immune infiltration and demonstrated that pyroptosis has an important impact on the tumor immune microenvironment. BRAF mutations could indirectly modulate the tumor immune microenvironment by modulating the pyroptosis-related signaling pathways (Erkes et al., 2020). A previous study has revealed that GSDMD is associated with immune infiltration in various cancers, such as ACC, BLCA (Bladder Urothelial Carcinoma), SKCM (Skin Cutaneous Melanoma), LGG (Brain Lower Grade Glioma), PCPG (Pheochromocytoma and Paraganglioma), and UVM (Uveal Melanoma) (Qiu et al., 2021).

From the analysis results of the immune checkpoint, we found that immune checkpoint LAG3 was significantly expressed in ACC, and the expression level of G4 patients was higher than that of G2 and G3 patients. In addition, we found that LAG3 was positively correlated with NLRC4 and GSDMB in five prognostic PRGs. These results suggest that NLRC4 and GSDMB can be used not only as prognostic characteristic genes of ACC patients but also as potential immunotherapeutic targets of ACC. However, further experimental verification is still needed. The research of Quentin Lecocq (Lecocq et al., 2020) revealed that combinatory treatments that incorporate the blockade of LAG-3 are viewed as a promising approach to improving current immunotherapies. As mentioned earlier, pyroptosis can not only promote the generation and development of cancer but also inhibit cancer. It is feasible to apply pyroptosis in the treatment of cancer, but few studies have been validated in clinical trials. Because the molecular mechanisms of pyroptosis and ACC have not been fully explained, we cannot assume here the therapeutic effect of pyroptosis on ACC, but there is no doubt that pyroptosis is closely related to ACC, and by observing pyroptosis factors, we can conveniently understand the patient’s cancer condition, therapeutic effect, and prognosis.

We validated CASP9 expression in ACC using qRT-PCR, Western blotting, and immunohistochemical staining methods. CASP9 is differentially expressed in normal tissues and ACC and is involved in the construction of prognostic models. CASP9 is also an important independent prognostic factor and is closely related to the level of immune infiltration. The above conclusions were validated by the results of qRT-PCR, Western blotting, and immunohistochemical staining experiments, which provided strong evidence for the above conclusions.

Does CASP9 also play a role in other cancers? CASP9 is involved in many cellular processes, caspase-9 is the initiator caspase of apoptosis, and is an important therapeutic target for various apoptosis-related diseases (Kim et al., 2015). What needs to be mentioned is, caspase (cysteinyl aspartate specific proteinase) is an endogenous aspartate proteolytic enzyme containing cysteine, which is an important gene family for organisms to maintain homeostasis by regulating cell death and inflammation. Pyroptosis is mediated by caspase, in which the classical pathway of pyroptosis is mediated by caspase-1, and the non-classical pathway of pyroptosis is mediated by caspase-4, caspase-5 and caspase-11. CASP9 is closely related to many cancers such as head and neck squamous cell carcinoma and breast cancer. Saman Sargazi's experiments demonstrated that CASP9 rs4645981 and rs1052571 polymorphisms are associated with overall cancer risk (Sargazi et al., 2021). What’s more, miR-182-5p inhibition can reduce the viability of MCF-7 (human breast cancer cell line) cells because of apoptosis induction, probably through the upregulation of CASP9 (Sharifi and Moridnia, 2017). In comparison with normal lung tissues, CASP9 gene expression was obviously downregulated in 44.4% (36/81) NSCLC (non-small cell lung cancer) tissues, which suggested the association between the CASP9 gene and NSCLC oncogenesis (Lou et al., 2007).

There are some deficiencies in our study. All analyses were performed using TCGA ACC queues, preferably with GEO queues for validation. In addition, *in vivo* and *in vitro* experiments are needed to further confirm our results. Moreover, how to interact between pyroptosis genes still needs further study. We would like to have a complete summary and discussion of the role of CASP9 in ACC, but unfortunately, there is a lack of relevant studies at present, which is our limitation and the direction of the next step in our plan.

In summary, we conducted a general and systematic bioinformatics analysis of ACC patients and identified the pyroptosis-related prognostic gene characteristic containing five genes (CASP3, CASP9, GSDMB, GSDMD, and NLRC4). CASP9 was identified as an independent prognostic factor of ACC patients. At the same time, we analyzed the correlation between PRGs and immune infiltration and immune checkpoints and found two genes (NLRC4 and GSDMB) that may be potential therapeutic targets. QRT-PCR, Western blot, and immunohistochemical staining were subsequently performed to verify mRNA and protein expression of the independent prognostic factor CASP9. However, there has been no phased breakthrough in the study of pyroptosis in adrenocortical carcinoma, and it may be meaningful to study its underlying mechanism in the future. Our results provide new prognostic markers for ACC patients, and we believe that these works will also help to investigate the mechanism of pyroptosis in ACC. The study on the prognostic value of PRGs lays a foundation for future mechanism research. We are more likely to make further breakthroughs in the study of ACC only if the molecular mechanisms are investigated clearly. We believe that genes such as CASP9 have the potential to play a role in further studies of ACC, which is our limitation and the direction of our next step. We expected our research could help ACC patients and provide reference significance for subsequent research in this field.

## 5 Conclusion

In summary, we identified pyroptosis-related prognostic gene signatures in ACC patients containing five genes (CASP3, CASP9, GSDMB, GSDMD, and NLRC4). Independent factor CASP9 was positively correlated to B cells and TMB. Our study also revealed the correlation between PRGs and immune checkpoints; NLRC4 and GSDMB may be considered potential therapeutic targets in the future. The results of qRT-PCR, Western blot, and immunohistochemical staining verified the mRNA expression levels of CASP9 in adjacent normal tissues and ACC tissues. We expected our study could provide further exploration direction and reference significance for subsequent research in this field.

## Data Availability

The datasets presented in this study can be found in online repositories. The names of the repository/repositories and accession number(s) can be found in the article/Supplementary Material.
