# Supplementary material for: The signature of pyroptosis-related gene prognostic and immune microenvironment in adrenocortical carcinoma
Source: Front Mol Biosci. 2023 Feb 24;10:1131402. doi: 10.3389/fmolb.2023.1131402 (PMC9998516; doi:10.3389/fmolb.2023.1131402)
Supplement: Supplementary file 1 [file Table1.DOCX]

Supplementary Material

**Supplementary Figure S1.** (A) protein-protein interaction (PPI) of PRGs. (B) the correlation network contains all PRGs.


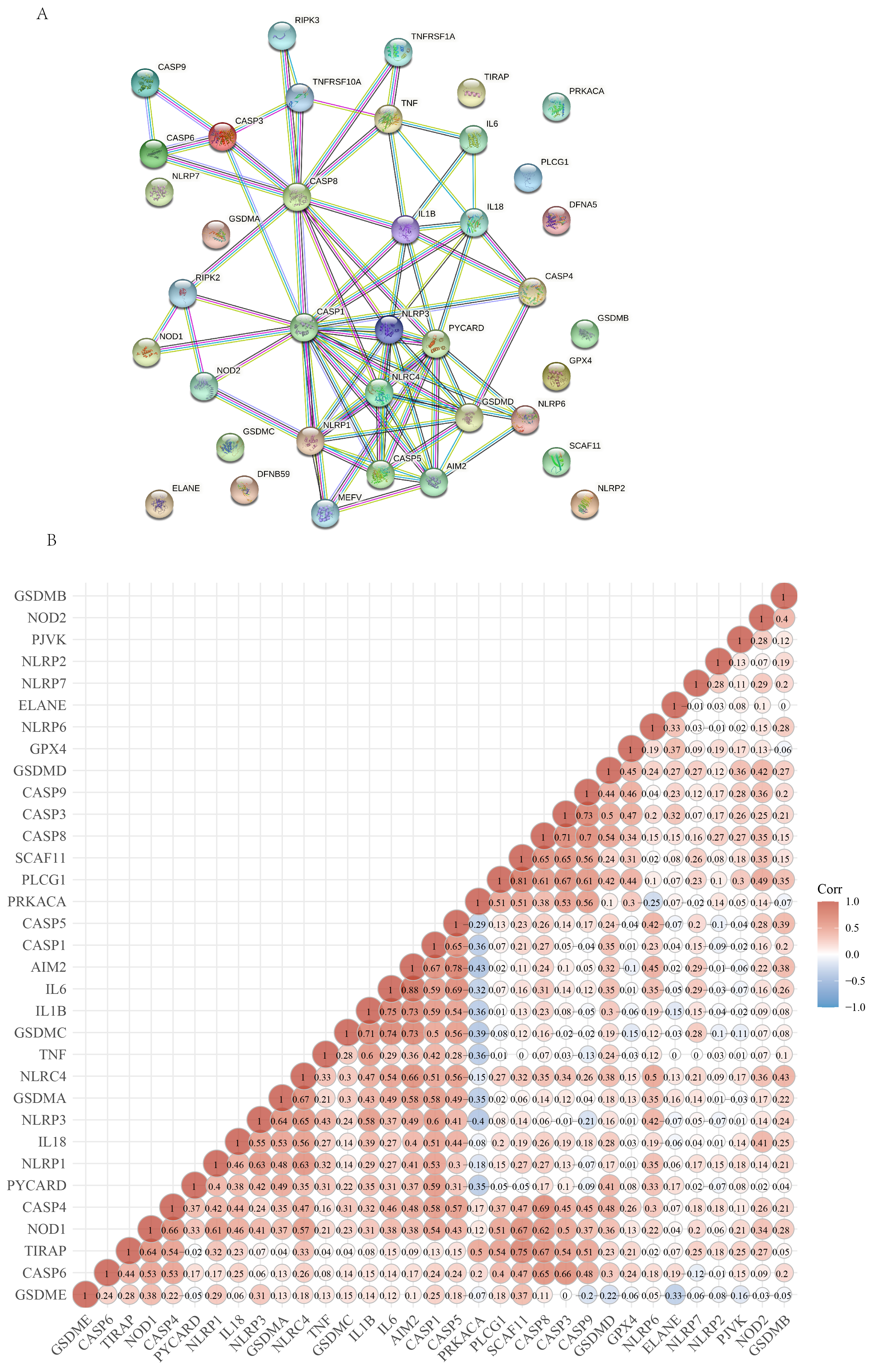


**Supplementary Table S1.** The clinical characteristics of Adrenocortical carcinoma patients in the TCGA cohort.

| Clinical characters | Number |
| --- | --- |
| Gender  Male  Female | 31  48 |
| Age |  |
| Mean (SD) | 46.7 |
| Median [MIN, MAX] | 49[14,77] |
| TNM stage  I  II  III  IV | 9  37  16  15 |
| pT_stage  T1  T2  T3  T4 | 9  42  8  18 |
| pN_stage  N0  N1 | 68  9 |
| pM_stage  M0  M1 | 62  15 |

**Supplementary Table S2.** 33 PRGs from previous studies.

| Genes | Full-names |
| --- | --- |
| AIM2 | Absent in melanoma 2 |
| CASP1 | cysteine-aspartic acid protease-1 |
| CASP3 | cysteine-aspartic acid protease-3 |
| CASP4 | cysteine-aspartic acid protease-4 |
| CASP5 | cysteine-aspartic acid protease-5 |
| CASP6 | cysteine-aspartic acid protease-6 |
| CASP8 | cysteine-aspartic acid protease-8 |
| CASP9 | cysteine-aspartic acid protease-9 |
| ELANE | elastase, neutrophil expressed |
| GPX4 | glutathione peroxidase 4 |
| GSDMA | gasdermin A |
| GSDMB | gasdermin B |
| GSDMC | gasdermin C |
| GSDMD | gasdermin D |
| GSDME | gasdermin E |
| IL18 | interleukin 18 |
| IL1B | interleukin 1 beta |
| IL6 | interleukin 6 |
| NLRC4 | NLR family CARD domain containing 4 |
| NLRP1 | NLR family pyrin domain containing 1 |
| NLRP2 | NLR family pyrin domain containing 2 |
| NLRP3 | NLR family pyrin domain containing 3 |
| NLRP6 | NLR family pyrin domain containing 6 |
| NLRP7 | NLR family pyrin domain containing 7 |
| NOD1 | nucleotide binding oligomerization domain containing 1 |
| NOD2 | nucleotide binding oligomerization domain containing 2 |
| PJVK | pejvakin/deafness, autosomal recessive 59 |
| PLCG1 | phospholipase C gamma 1 |
| PRKACA | protein kinase cAMP-activated catalytic subunit alpha |
| PYCARD | PYD and CARD domain containing |
| SCAF11 | SR-related CTD associated factor 11 |
| TIRAP | TIR domain containing adaptor protein |
| TNF | tumor necrosis factor |
